# Variations in Anxiety and Related Psychiatric Comorbidity Levels Among Youths With Individual Diagnoses of Autism Spectrum Disorder or Attention Deficit Hyperactivity Disorder and Those With Both Diagnoses

**DOI:** 10.7759/cureus.41759

**Published:** 2023-07-12

**Authors:** Michael Wu, Ernesto Joubran, Divya Kumar, Nasser D Assadi, Hoang Nguyen

**Affiliations:** 1 Medicine, Nova Southeastern University Dr. Kiran C. Patel College of Osteopathic Medicine, Fort Lauderdale, USA; 2 Osteopathic Medicine, Nova Southeastern University Dr. Kiran C. Patel College of Osteopathic Medicine, Fort Lauderdale, USA; 3 Basic Sciences, Nova Southeastern University Dr. Kiran C. Patel College of Osteopathic Medicine, Clearwater, USA

**Keywords:** medicine-pediatrics, child and youth mental health, attention deficit hyperactivity disorder (adhd), autism spectrum disorder and emotion, clinical anxiety

## Abstract

Children with attention deficit hyperactivity disorder (ADHD) or autism spectrum disorder (ASD) individually and those with co-occurring ADHD and ASD experience higher rates of total anxiety and psychiatric comorbidities such as gender dysphoria and locomotor skills compared to their typically developing (TD) peers. In this study, it was hypothesized that youth with comorbid ADHD and ASD would experience higher levels of overall anxiety, specifically separation, generalized, and social anxiety. A literature review of relevant studies published from 2007 to 2020 was performed, with a search involving key terms such as “Anxiety,” “ADHD” and “ASD'". It was discovered that individuals with ADHD or ASD had higher levels of anxiety compared to their peers. Furthermore, children who have co-occurring ADHD and ASD had more serve levels of anxiety than children with an individual diagnosis of ADHD or ASD. Children with ASD, ADHD, and co-occurring ADHD and ASD had a higher prevalence of gender dysphoria and impaired locomotor skills, which lead to higher levels of psychiatric comorbidities seen in this population. It can be hypothesized psychiatric comorbidities could also have implications for the high anxiety levels seen in this population but further research is needed to confirm this.

## Introduction and background

Attention deficit hyperactivity disorder (ADHD) is a common neurological, and developmental comorbid disorder among youth with autism spectrum disorder (ASD) [1]. However, the degree to which comorbid anxiety is comparable in these syndromes is still not well-understood. It has been suggested that comorbidity leads to a more severe deficiency due to the contributory effects of one disorder superimposed on a second one. Nonetheless, the co-occurrence of these disorders does not result in a unique condition [2].

Over the past decade, the number of patients being treated for ADHD has increased worldwide. There has been a rise in patients with ADHD in European countries such as the Netherlands, Denmark, and Spain, while the rates in most European countries are much lower when compared to the US. The use of prescription medications has increased by 30-85% in children who have ADHD along with other comorbidities, such as ASD or pervasive developmental disorder (PDD) [3,4]. There is also a growing trend of children with ASD exhibiting other neurodevelopmental disorders, especially ADHD and anxiety [4,5].

With regard to the response to treatment, there appears to be a consensus among researchers. This entails that comorbidity is qualitatively diverse when compared to the combination of single disorders. Research findings pertaining to patients of various ages with diverse combinations of ASD and ADHD have reported a variance in the rates of co-presentation of anxiety in patients affected by multiple disorders versus patients affected by a single disorder [2].

ADHD has been further classified into three subtypes: ADHD-predominantly inattentive (ADHD-PI), ADHD-predominantly hyperactive-impulsive (ADHD-HI), and ADHD-combined type (ADHD-C). Motor deficits in children with ADHD are well recognized extensively in the ADHD-related literature. The research predominantly suggests that the movement skills of children with the disorder are significantly distinctive in comparison to peers without ADHD [6]. In addition, several studies have reported that non-comorbid ADHD occurs in only 13-32.3% of cases and that patients with ADHD have multiple comorbid disorders. Comorbidity can refer to both the co-occurrence of disorders temporally separated from ADHD or concurrent with ADHD. The rate of co-occurrence of ADHD and other disorders varies, as do the prevalence rates of these individual disorders [7-9].

Research also shows a prevalence of ADHD in certain psychiatric comorbidities. It is especially high in individuals with mood disorders (22%), anxiety disorders (34%), substance use disorders (11%), and most behavioral disorders (15%) [10]. In a study of about 40,000 adults, ADHD patients manifested a four to nine times higher prevalence of anxiety, depression, various other behavioral/mood disorders, and substance use disorders [10]. The strongest predictor of poor quality of life in adults diagnosed with ADHD is associated with the co-occurrence of both anxiety and depression [5]. According to the Mini International Neuropsychiatric Interview (MINI), patients with generalized anxiety disorder (GAD) make up 25% of patients with comorbid ADHD and 8% of those with ASD [5,11].

Several studies have found that comorbid anxiety disorders can even alter cognitive test performances in patients with ADHD. While there have been some failures in replicating these tests, anxiety disorders can affect physiological arousal, apprehension, and tension [12]. Communication and social skills in these patients have also been studied and evaluated. The research has shown that children with ASD present difficulties in social behavior. Those with combined ASD and ADHD showed a significant deficit in social communication and relationships [13].

Finally, neurodevelopmental conditions such as ASD and ADHD are found more commonly in transgender individuals [14]. There has also been debate about certain conditions being more common in patients who suffer from gender dysphoria. In addition to ADHD, ASD may be more prevalent among individuals with gender dysphoria. Attention and social deficits can affect the assessment of gender dysphoria [5,14]. Concerning transgender healthcare services, there is currently a greater global incidence of the perceived number of transgender patients, based on the fact that a higher number of these individuals are seeking healthcare services compared to the past decades. Moreover, there is overwhelming evidence to indicate a high prevalence of depression and anxiety among individuals with gender dysphoria [15]. 

In clinical settings, anxiety is encountered at an increasing rate during school years in patients with ASD. Both children with ADHD or ASD individually and those with comorbid ADHD and ASD experience higher rates of total anxiety when compared to their typically developing (TD) peers [16]. Youths with ASD often experience separation anxiety, social anxiety, and generalized anxiety symptoms, while those with ADHD typically report heightened separation and generalized anxiety [17,18]. ADHD and ASD may confer differential and conjoint risk for specific anxiety symptoms in youth.

This literature review aims to investigate the possible associations between psychiatric comorbidities and anxiety in patients with individual diagnoses of ADHD or ASD and those with co-occurring ADHD and ASD. It is hypothesized that individuals with ASD, ADHD, or co-occurring ADHD and ASD will face higher levels of anxiety and psychiatric comorbidities that can be directly attributed to their disease. This is the first study to date to examine the relative impact of comorbid ASD and ADHD on specific anxiety symptoms in youths.

This article was previously presented as a meeting abstract at the 2019 International Society for Autism Research (INSAR) Meeting on May 2, 2019.

## Review

Materials and methods

A search was carried out on the databases PubMed, Scopus, and Medline for published and peer-reviewed literature addressing differing anxiety levels in individuals with ASD, ADHD, and co-occurring ASD and ADHD. We also used the same databases to find various published peer-reviewed articles that address various psychiatric comorbidities seen in children with ASD, ADHD, and those with co-occurring ASD and ADHD. Our inclusion criteria were as follows: articles involving children with a diagnosis of ASD, ADHD, or co-occurring ADHD and ASD. The search yielded a total of eight articles that addressed various levels of anxiety in our target population as well as psychiatric comorbidities such as alterations in locomotor skills and gender dysphoria. 

Results

A Summary of the literature review is presented in Table 1 below.

**Table 1:** Data compiled and results gathered ADI-R = Autism Diagnostic Interview-Revised; ADHD = Attention Deficit Hyperactivity Disorder; ADOS = Autism Diagnostic Observation Schedule; ANCOVAs = Univariate Analyses of Covariance; ANOVA = Analysis of Variance; ASD = Autism Spectrum Disorder; AQ = Autism Spectrum Quotient; BAARS-IV = Barkley Adult ADHD Rating Scale-IV; BDI-II = Beck Depression Inventory II; BSID-III = Bayley Scales of Infant and Toddler Development-Third Edition; CARS-2 = Childhood Autism Rating Scale- Second Edition; CPT-II = Conners’ Continuous Performance Test II; CSI-4 = Child Symptom Inventory-4; DD = Depressive Disorder; DICA-P = Diagnostic Interview for Children and Adolescents; DISCO-10 = Diagnostic for Social and Communication Disorders-Tenth Edition; DIVA = Diagnostic Interview for ADHD in Adults; DPCR = Danish Psychiatric Central Registrar; DSM-IIIR = Diagnostic and Statistical Manual of Mental Disorders–Third Edition; DSM-IV = Diagnostic and Statistical Manual of Mental Disorders–Fourth Edition; DSM-5 = Diagnostic and Statistical Manual of Mental Disorders–Fifth Edition; ECBQ = Early Childhood Behavior Questionnaire; GAD = Generalized Anxiety Disorder; GD = Gender Dysphoria; GMDQ = Gross Motor Development Quotient; IBQ-R = Infant Behavior Questionnaire-Revised; ICD-10 = International Classification of Diseases-Tenth Revision; MASC = Multidimensional Anxiety Scale for Children; MINI interview = Mini International Neuropsychiatric Interview; OR = Odds Ratio; Rx = Prescription; SAD = Separation Anxiety Disorder; SD = Standard Deviation; SE = Standard Error; SRS-2 = Social Responsive Scale-Second Edition; SSRT = Stop-Signal Reaction Time; SUD = Substance use Disorder; TGMD-2 = Test of Gross Motor Development- Second Edition; WISC-IV = Weschler Intelligence Scale for Children-Fourth Edition; WPPSI-III = Weschler Preschool and Primary Scale of Intelligence-Third Edition; 95% CI = 95% Confidence Interval

Authors	Purpose of study	Participants	Study design/measures	Results
Jensen et al. (2014) [9]	Assess the presence of comorbid developmental disorders with ADHD	825 individuals diagnosed with ADHD between 4-17 years of age. 79.4% males; 20.6% females. Male diagnosis (mean = 9.6, SD = 3.4), female diagnosis (mean = 10.7, SD = 3.9 years)	ICD-10 criteria; general intelligence and neurophysiological testing; teacher-reported and parent-reported behavior and functioning information collected	1) Anxiety disorders+ADHD were identified in late childhood or early adolescence. The most common comorbid disorders in ADHD patients were conduct disorders (16.5%), specific developmental disorders of language, learning, and motor skills (15.4%), autism spectrum disorder (12.4%), and intellectual disability (7.9%). 2) Males were diagnosed with anxiety at significantly lower rates (1% vs. 2.1%; OR:0.60; 95%CI: 0.40-0.80, p<0.01). 3) After adjusting for age, females were at a higher risk for comorbid affective disorders (1.3% vs. 4.5%, p<0.01), anxiety disorders (1% vs. 2.1%, p<0.01), reactions to severe stress (2.2% vs 6.7%, p<0.01), and eating disorders (0.1% vs. 1.3%, p<0.01). 4) After adjusting for age, males were at a greater risk of for comorbid autism spectrum disorder (13.3% vs. 9.2%, p<0.01), conduct disorder (17.2% vs. 13.7%, p<0.01), tic disorders (5.3% vs. 2.9%, p<0.01), and specific disorders of development (16.1% vs. 12.8%, p<0.01). 5) Low reporting of anxiety symptoms (1.3%) and affective disorders (2%) compared to previous studies is likely due to interpretation as a reaction to life stressors, not as a disorder, as theorized by the authors. 6) ADHD/tic disorders were strongly related to anxiety disorders
Pan et al. (2009) [6]	Compare movement and locomotor skills between those with ADHD and ASD	91 children, age 6-10 years, average IQ. ASD (n = 91); ADHD (n = 29); typically developing children (n = 34)	TGMD-2 (evaluate locomotor skills); GMDQ; DSM-IV; psychiatrist diagnosis of ADHD; physical education and classroom teacher reported data	1) Among the ADHD, ASD, and control groups, there were significant differences in galloping [ꭓ2 (14, n = 91) = 43.08, p<0.001], hopping [ꭓ2 (20, n = 91) p = 43.08
Guttmann-Steintmetz et al. (2010) [2]	Examine differences in co-occurring anxiety, specifically GAD and SAD, between different neurobehavioral syndromes	ASD + ADHD (n = 74), ASD only (n = 130), and ADHD only (n = 59)	Parent and teacher versions of DSM-IV; CSI-4; DICA-P; DSM-IIIR and DSM-IV criteria; ADOS	1) Comorbid ASD and ADHD showed generally higher mother-rated GAD scores than the ADHD-only group. 2) ASD social skill deficits had low to moderate association with ratings of GAD and SAD symptoms, while ASD communication skills are minimally associated with GAD and SAD. 3) ASD+ADHD groups had a more evident relationship to GAD and SAD based on teacher ratings
Shepard et al. (2019) [4]	Evaluate early-life predictors of mid-childhood ASD and anxiety symptoms when compared to ASD symptoms	Infants (n = 104) at high and low familial risk for ASD took part in research assessments at 7, 14, 24, and 38 months and 7 years of age	Parent-reported symptoms of ASD, ADHD, and anxiety; IBQ-R; ECBQ; SRS-2	1) Fear and Activity were positively correlated with statistical significance (r = .54, p < .001). 2) There was no significant correlation between shyness and inhibitory control (r = .14, p = .162). 3) Inhibitory control was a significant predictor of ADHD symptoms but not anxiety after controlling for ASD symptoms (standardized beta: -0.29; 95%CI: 0.49-0.09, p = 0.005). 4) Population at High familial risk for ASD is also vulnerable to anxiety, ADHD, and other developmental outcomes. ASD and anxiety are difficult to distinguish in early childhood
Wood and Gadow (2010) [11]	Examine the pathology of anxiety disorder in youth on the autism spectrum as well as social skill deficits and repetitive behaviors exacerbated by ASD	Research articles including key words autism spectrum disorder, child anxiety disorders, comorbidity, differential diagnosis	DSM-IV	1) Several studies on children with ASD have found strong links between high anxiety and greater severity of ASD symptoms, including sensory symptoms. 2) Anxiety symptoms and disorders are generally associated with significant functional impairment. 3) Higher anxiety was associated with greater total ASD symptoms, even when controlling for intellectual impairment, social maladjustment, and degree of speech impairment. 4) Although many youths with ASD present high rates of anxiety symptoms, relatively little can be said about the nature or origin of these behaviors or their differentiation into multiple anxiety syndromes
Ruf et al. (2016) [12]	Evaluate the effect of individual differences in trait anxiety on cognitive test performance in adolescents	221 adolescents (79 females, 142 males), including 98 diagnosed based on DSM-IV attention-deficit/hyperactivity disorder combined subtype (DSM-IV 314.01) and 123 healthy control subjects, between ages 12 and 18 years (mean = 15.3)	MASC; DMS-IV; CPT-II	1) Trait anxiety is seen as a “protective” characteristic for ADHD-diagnosed adolescents in which they had less abnormal performances on CPT-II attention tests. 2) Only SSRT score had a main effect on MASC-measured trait anxiety (regression standardized β = −.202, p = 0.043). 3) ADHD CPT–II hit reaction time was only correlated with CPT–II variability scores (r = .411, p < .001); ADHD CPT–II omissions, variability, and response style were highly intercorrelated in ADHD patients (from r = 0.291, p = 0.005 to r = 0.612, p<0.001). 4) Pearson correlation found a significant association between BDI–II score and SSRT (r = −.145, p = 0.039) 5) MASC scores showed a statistically significant relation to hyperactive/impulsive symptom counts (r = 0.197, p = 0.05) 6) Greater hyperactive/impulsive symptom counts were correlated with higher trait anxiety
Thrower et al. (2019) [14]	Examine the prevalence of ASD and ADHD in individuals with gender dysphoria	English peer-reviewed publications were included, which included 179 studies. After applying exclusion criteria, 22 studies examined the prevalence of ASD or ADHD in people with GD. A further 8 studies examined the opposite; the prevalence of GD in people with ASD	AQ; DISCO-10	1) Positive screening tests for ASD in patients with GD range from 6-68%. 2) 45-68% of youth with GD were found to have autistic symptoms based on the evaluation that they were having “difficulty relating to their peers.” 3) In the studies examined, the prevalence of prior ADHD in patients with GD ranged from 4.3-20.4%
Salley et al. (2015) [13]	Evaluate communication and social interaction impairment in youth diagnosed with ASD and/or ADHD	209 youth aged 3 to 18 years, with a mean age of 7.39 years (SD = 3.84 years). Youth were primarily White (65%), followed by Biracial (9%), Black or African American (8%), Hispanic (8%), and Other (10%)	CARS-2; ADI-R; ADOS; WPPSI-III; WISC-IV; BSID-III; DSM-IV; DSM-5	1) There was a significant association between age and communication or social interaction ADOS score (Pearson’s r = 0.144; p = 0.039). 2) Mean ADOS score difference by group: No Diagnosis (mean = 4.902; SE = 0.570; 95%CI: 3.777-6.027); ADHD (mean = 5.452; SE = 0.740; 95%CI: 3.992-6.912); ASD+ADHD (mean = 10.674; SE = 0.689; 95%CI: 9.316-12.032); ASD (mean = 12.858; SE = 0.428; 95%CI: 12.013-13.702) 3) Study provides novel evidence for utilization of the ADOS for distinguishing ADHD only youth vs. ASD alone or co-occurring ASD+ADHD

Discussion

We conducted a literature review on the differing levels of anxiety and psychiatric comorbidities in children with ADHD, ASD, and co-occurring diseases. Clinicians have recognized an increased prevalence of anxiety and psychiatric comorbidities such as gender dysphoria and impaired locomotor skills in children with various diagnoses. The levels of anxiety and psychiatric comorbidities appear to be intertwined as cases of gender dysphoria have been observed to cause increased levels of anxiety [14]. Anxiety also potentially stems from limited motor skills in children with ADHD, ASD, or co-occurring ADHD and ASD as they are limited in their interaction capabilities with peers [6]. Although children with ADHD, ASD, and co-occurring ADHD and ASD may not directly have anxiety stemming from their diagnosis, the associated psychiatric comorbidities may lead to increasing their anxiety levels.

In a study conducted by Guttmann-Steintmetz et al., the particular focus was on anxiety symptoms present in boys and in groups with ASD, ADHD, and ADHD + ASD [2]. They also explored symptoms of chronic multiple tic disorder (CMTD). They found that mother’s ratings did not vary between groups of ASD + ADHD and CMTD + ADHD, but the teacher’s ratings showed a higher differentiation in the “irritable” and “acts restless” categories for those with ASD + ADHD in comparison to CMTD + ADHD [2]. Whether they had comorbid ASD or CMTD, individuals with ADHD were found to have higher levels of GAD and separation anxiety disorder (SAD) [2]. CMTD encompasses muscle spasms and involuntary movement, which is a factor that differentiated the severity of SAD symptoms, but differences between SAD and GAD symptoms were not further evaluated [2]. Additional research investigating differences in levels of SAD and GAD as well as specific subcategories can provide further insights.

Identification of ADHD and ASD symptoms can vary based on age and early-life atypicality. Recognizing these symptoms may aid in the prediction of the presence of ADHD, ASD, and anxiety [4]. Shephard et al. noted that activity level and inhibitory control in early life are associated with ADHD, but not seen in ASD individuals or those affected by anxiety [4]. Their findings indicate that some atypicality seen in ADHD is not present in ASD, which suggests that these conditions do not arise from a common source [4]. However, mid-childhood ASD and anxiety were commonly associated with fearfulness and shyness. In individuals with ASD, a high frequency of anxiety is detected due to the stress and anxiety associated with social interactions [4]. Identifying early-life markers of ASD and ADHD as individually evolving conditions has been well looked into with hopes of establishing a deeper understanding of disease and early markers.

Salley et al. reported that people with ASD demonstrated the highest communication and social interaction deficit while those with ASD + ADHD had the second highest deficiency [12]. Those who were diagnosed with ASD had a greater communication impairment than those with ADHD [12]. These findings may correlate with signs of anxiety such as social phobia. Overlapping symptoms between ASD and anxiety may be suggestive of the increased level of social interaction deficits in ASD and ASD + ADHD groups, as compared to individuals diagnosed with ADHD [11]. In ASD individuals, communication deficits were proven to be an overlapping symptom that has links to social anxiety disorders. All of these could lead individuals who have ASD to have increased separation anxiety, especially children who lack social interaction and communication skills. These findings suggest that individuals with ASD are more likely to have social phobia and anxiety symptoms.

A study conducted by Jensen et al., found that female children with ADHD had a higher prevalence of anxiety disorder than their male counterparts [9]. The study also identified a lower rate of ADHD children having a co-occurring anxiety disorder, which the authors attributed to clinicians identifying the symptoms as a reaction to the stressful situations encountered by these individuals [9]. They also found that the development of anxiety was more likely to be identified in the adolescent stage of development. This could potentially be attributed to the increased social interaction that comes with progressing age, which could induce more stressful situations. The results of this study support the idea that children with ADHD, ASD, and co-occurring ADHD and ASD face higher levels of anxiety and comorbid psychiatric conditions. 

Pan et al. evaluated the relationship between motor skills in those with ASD and ADHD among children in Taiwan [6]. Motor skills performance in children with ASD and ADHD was noted to be worse than their TD peers in the categories of gross motor development, locomotor, and object control skills [6]. These findings illustrate the motor deficits children with ASD and ADHD face, which could induce limitations in socialization and interaction with other children and lead to higher levels of anxiety. Further, studies into the effects of impaired motor skills in children with ASD and ADHD are needed to understand the implications motor disorders have on children's anxiety.

Although there is evidence of children with ADHD, ASD, and co-occurring ADHD and ASD having higher rates of gender dysphoria the evidence is limited. A review by Thrower et al. found that children with ASD face gender dysphoria at a rate four times higher than the general population. They also found an increased rate of GD in individuals with ADHD as well [14]. This study was able to clearly delineate that children with gender dysphoria have higher levels of ADHD and ASD. The systemic review also linked individuals with gender dysphoria to having higher levels of anxiety [14]. Although this study lends support to the idea that children with ADHD, ASD, and co-occurring ADHD and ASD face higher levels of anxiety and comorbid psychiatric conditions, further research is needed to truly delineate the association.

Children with ADHD face higher levels of anxiety than others with estimated rates of co-occurring anxiety being between 18% - 32% of children [12]. Ruf et al. analyzed the cognitive effects of these increased anxiety rates in children with ADHD. They found anxiety to be a protective factor when analyzing the cognitive activity and function of these individuals [12]. They also found that children with ADHD and co-occurring anxiety had a less abnormal performance on several theoretically distinct indices of the CPT-II attention test [12]. This study is unique in that it aligns with our hypothesis that children with ADHD face higher levels of anxiety, but it identifies anxiety as a positive factor that aids children with ADHD by increasing their cognitive performance.

Anxiety is an extensively studied disorder, with a 25% coexistence rate in those with ADHD [7]. Unlike most studies that are in accordance with this finding, Joshi et al. reported that the prevalence of anxiety in ADHD is relatively lower [7]. This may be due to differences in diagnostic criteria as Joshi et al. defined a clinically relevant anxiety syndrome as the presence of two or more anxiety disorders [7]. Many clinicians attribute anxiety symptoms in children with ADHD to life stressors and therefore may consider it as stress rather than anxiety. This explains its low reported levels. Another disorder that is not extensively mentioned in this study, as compared to previous studies, is tic disorder, which may again be due to a narrow definition of its comorbidity or due to the onset of tic after ADHD diagnosis. Previous studies have found a strong relationship between ADHD/tic disorder and anxiety. Guttmann-Steintmetz et al. stated that in accordance with various other sources, male children with ADHD have similarly reported GAD and SAD symptoms regardless of comorbid ASD or CMTD [2].

Our findings suggest an association between those with ADHD, ASD, or co-occurring diseases and higher levels of anxiety and psychiatric comorbidities. We believe that the evidence presented will encourage various health professionals to become more vigilant and aware of these associated symptoms in children with confirmed ADHD, ASD, and co-occurring diagnoses. With both ADHD and ASD having an uncertain etiology, it is hard to truly understand why individuals with ADHD and ASD have an increased prevalence of associated anxiety and psychiatric symptoms. This study has a few limitations, including the varying criteria applied for anxiety diagnosis. Some clinicians can attribute anxiety to stressful situations instead of clinical anxiety disorder, which could contribute to a lower overall anxiety diagnosis in this population. Further research is needed to understand the causative factors that link higher levels of anxiety and associated psychiatric symptoms in children with ADHD, ASD, and co-occurring ADHD and ASD. 

## Conclusions

This literature review aimed to evaluate the possible linkage between anxiety and psychiatric comorbidities in individuals with ASD, ADHD, and co-occurring ADHD and ASD. We have found evidence indicating that individuals with ADHD, ASD, or co-occurring ADHD and ASD display higher levels of anxiety and psychiatric comorbidities such as impaired locomotor skills and gender dysphoria. Our findings provide deeper insights into the association between anxiety and comorbid psychiatric symptoms in individuals diagnosed with ADHD, ASD, or co-occurring ADHD and ASD. Further research is needed to truly delineate the associations seen between anxiety and comorbid psychiatric disorders in those with ASD and ADHD as well as aid in gaining a deeper understanding of the treatment models required for treating these conditions.

## References

[1] Antshel KM, Zhang-James Y, Faraone SV (2013). The comorbidity of ADHD and autism spectrum disorder. Expert Rev Neurother.

[2] Guttmann-Steinmetz S, Gadow KD, DeVincent CJ, Crowell J (2010). Anxiety symptoms in boys with autism spectrum disorder, attention-deficit hyperactivity disorder, or chronic multiple tic disorder and community controls. J Autism Dev Disord.

[3] Dalsgaard S, Nielsen HS, Simonsen M (2013). Five-fold increase in national prevalence rates of attention-deficit/hyperactivity disorder medications for children and adolescents with autism spectrum disorder, attention-deficit/hyperactivity disorder, and other psychiatric disorders: a Danish register-based study. J Child Adolesc Psychopharmacol.

[4] Shephard E, Bedford R, Milosavljevic B (2019). Early developmental pathways to childhood symptoms of attention-deficit hyperactivity disorder, anxiety and autism spectrum disorder. J Child Psychol Psychiatry.

[5] Pehlivanidis A, Papanikolaou K, Mantas V (2020). Lifetime co-occurring psychiatric disorders in newly diagnosed adults with attention deficit hyperactivity disorder (ADHD) or/and autism spectrum disorder (ASD). BMC Psychiatry.

[6] Pan CY, Tsai CL, Chu CH (2009). Fundamental movement skills in children diagnosed with autism spectrum disorders and attention deficit hyperactivity disorder. J Autism Dev Disord.

[7] Joshi G, Wozniak J, Petty C (2013). Psychiatric comorbidity and functioning in a clinically referred population of adults with autism spectrum disorders: a comparative study. J Autism Dev Disord.

[8] White SW, Oswald D, Ollendick T, Scahill L (2009). Anxiety in children and adolescents with autism spectrum disorders. Clin Psychol Rev.

[9] Jensen CM, Steinhausen HC (2015). Comorbid mental disorders in children and adolescents with attention-deficit/hyperactivity disorder in a large nationwide study. Atten Defic Hyperact Disord.

[10] Fayyad J, Sampson NA, Hwang I (2017). The descriptive epidemiology of DSM-IV Adult ADHD in the World Health Organization World Mental Health Surveys. Atten Defic Hyperact Disord.

[11] Wood JJ, Gadow KD (2010). Exploring the nature and function of anxiety in youth with autism spectrum disorders. Clin Psychol Sci Prac.

[12] Ruf BM, Bessette KL, Pearlson GD, Stevens MC (2017). Effect of trait anxiety on cognitive test performance in adolescents with and without attention-deficit/hyperactivity disorder. J Clin Exp Neuropsychol.

[13] Salley B, Gabrielli J, Smith CM, Braun M (2015). Do communication and social interaction skills differ across youth diagnosed with autism spectrum disorder, attention-deficit/hyperactivity disorder, or dual diagnosis?. Res Autism Spectr Disord.

[14] Thrower E, Bretherton I, Pang KC, Zajac JD, Cheung AS (2020). Prevalence of autism spectrum disorder and attention-deficit hyperactivity disorder amongst individuals with gender dysphoria: a systematic review. J Autism Dev Disord.

[15] Bhatt N, Cannella J, Gentile JP (2022). Gender-affirming Care for Transgender Patients. Innov Clin Neurosci.

[16] Bauermeister JJ, Shrout PE, Ramírez R (2007). ADHD correlates, comorbidity, and impairment in community and treated samples of children and adolescents. J Abnorm Child Psychol.

[17] van Steensel FJ, Bögels SM, Perrin S (2011). Anxiety disorders in children and adolescents with autistic spectrum disorders: a meta-analysis. Clin Child Fam Psychol Rev.

[18] Tsang TW, Kohn MR, Efron D, Clarke SD, Clark CR, Lamb C, Williams LM (2015). Anxiety in young people with ADHD: clinical and self-report outcomes. J Atten Disord.

